# Prof. Chisolm's Rules in Administering Chloroform

**Published:** 1883-05

**Authors:** 


ARTICLE VIII.

### DR. CHISOLM'S RULES IN ADMINISTERING CHLOROFORM.

After an experience of thirty years of an active surgical practice, I still hold chloroform to be the best of anæsthetics for tedious operations, provided certain simple rules are adhered to in its administration. I can enumerate them in very few words:

1. I always, without a single exception, give a strong drink of whiskey, from one to two ounces, to every adult to whom I intend to administer chloroform. This is done a few minutes before they get on the operating table. Because I never omit this fundamental law, and in advance sustain the heart against the depressing effect of the anæsthetic, in not one of my 12,000 cases have I had to use, in a single instance, a hypodermic of whiskey. It is already in the stomach, should it be needed, and can do no harm if not required.

2. Always loose the neck and chest clothing so as to have no impediment to respiration.

3. Only administer chloroform in the recumbent posture with body perfectly horizontal and head on a low pillow, this pillow to be removed as the anæsthesia progresses.

4. Give chloroform on a thin towel folded in conical form with open apex so that the vapor, before inhalation, will be freeiy diluted with atmospheric air. In holding this cone over the face of the patient at some little distance from the nose, place the fingers under the borders of the cone for the double purpose of allowing air to enter freely, and also to prevent the chloroform liquid on the towel from coming in contact with the skin of the patient's face, and thereby avoid its blistering effects.

5. Should loud snoring occur, force up the chin. This manipulation, by straightening the air passages from the nose to the larynx, makes easy breathing. The forcible elevation of the chin is far better in every respect than pulling out the tongue. It is easier of application, more quickly done, requires no instruments, and is much more efficient in removing the impediment to respiration.

By always following these five simple rules I have had, so far, both safety and comfort in the administration of chloroform.

Possibly one very strong reason why I have been so successful in the administration of chloroform is, that as a specialist in eye surgery the inhaler must be removed from the nose before I commence the surgical manipulations. Besides, while operating, I have constantly in view both the color of the face and the respiration of the patient, which I consider even more important for the surgeon to observe than to feel the pulse. When surgeons are operating on distant parts of the body and cannot watch the administrator of chloroform, accidents are most apt to happen.—*Prof. Chisolm of the University of Maryland.*

